# Combined Synergetic Effect of Lipoconcentrate Fat Grafting, Nanofat Transfer, Platelet-Rich Plasma, Microneedling, and CO2 Fractional Laser for Plastic Regenerative and Esthetic Surgery and Cosmetic Care

**DOI:** 10.7759/cureus.44035

**Published:** 2023-08-24

**Authors:** Sarah Qari, Maryam Bader, Eyas Farran, Renad Borrah, Sherif Khamis, Ziyad Alharbi

**Affiliations:** 1 Plastic Surgery and Burn Unit, Dr. Soliman Fakeeh Hospital, Jeddah, SAU; 2 Plastic Surgery and Burn Unit, King Fahad General Hospital, Jeddah, SAU; 3 College of Medicine, Fakeeh College for Medical Sciences, Jeddah, SAU; 4 Clinical Sciences Department, Fakeeh College for Medical Sciences, Jeddah, SAU

**Keywords:** adipose-derived stem cells (ascs), microneedling, platelet-rich plasma (prp), lipoconcentrate, nanofat, skin care

## Abstract

The advancements in skin care methods and products show the rising interest in cosmetics. Recent studies emphasize the regenerative potential of fat grafting, platelet-rich plasma (PRP), microneedling, and carbon dioxide (CO_2_) fractional laser techniques. Combining these strategies into a protocol is yet to be explored. In this article, we demonstrate different types of fat grafts and their versatility in treating different facial problems found in our patient. This study evaluated the synergistic effect of lipoconcentrate and nanofat grafting, PRP, microneedling, and CO_2_ fractional laser to provide esthetic and regenerative facial skin care. This case report was conducted in Dr. Soliman Fakeeh Hospital, Saudi Arabia. Our case involved a 53-year-old woman who had traumatic facial injuries due to a car accident years ago that buried asphalt particles in her facial scars, causing bluish skin discoloration. She suffered from multiple deep atrophic scars in several areas on the left side of her face, causing asymmetry. She was treated using lipoconcentrate and nanofat grafting, followed by three PRP with microneedling sessions and then a final CO_2_ fractional laser session. The evaluation was based on the physician’s clinical assessment, image documentation, and patient satisfaction, which revealed significant improvement in skin appearance with respect to texture, color, symmetry, and overall health of the skin over a period of four months. The potentiality and efficacy of the combination therapy of lipoconcentrate, nanofat, PRP, microneedling, and CO_2 _fractional laser for skin rejuvenation and scar treatment showed promising results in this case report.

## Introduction

Patients often view facial scars and aging skin as stigmatizing and disfiguring. Additionally, treatment is difficult and time-consuming. For cosmetic enhancement and scar healing, regenerative and renewing treatments can be utilized. In cosmetic enhancement, the color, texture, and general health of the skin are typically considered, whereas, in the case of atrophic scars, lipofilling is typically the first step, followed by addressing esthetic concerns. Both patient types continue to experience negative emotions that adversely impact their social lives [1].

In recent decades, autologous lipofilling, which was pioneered by Coleman et al. [2], has become a routine technique in reconstructive and cosmetic plastic surgery. Fat grafting is an effective treatment for facial scars and rejuvenation of aging skin due to the regenerative properties of autologous fat tissue [3]. Individual case studies have demonstrated that autologous lipofilling can be used to treat post-burn scars [4]. The use of the patient’s own adipose tissue makes this an appealing alternative to synthetic products.

According to the technique of adipocyte harvesting and manipulation, various fat grafts exist. Macrofats are large fat particles used primarily for large-volume fat transfers, such as to the buttocks or breasts, but they can also be utilized for smaller fat transfers to the face, such as the cheekbones or lips. Due to a higher concentration of adipose-derived stem cells (ASCs), lipoconcentrates have superior regenerative properties and a lower volumizing effect than microfats. In an ideal situation, lipoconcentrates are applied to the face to prevent the appearance of visible lumps in areas such as the under-eye region, nasolabial folds, and forehead. Finally, nanofats, which are liquid fat that has been converted, improve the texture and color of the skin through regeneration without adding volume [1,3,5].

ASCs, which are multipotent mesenchymal/stromal stem cells, are essential to the regenerative capacity of adipose tissue. It is widely accepted that these cells can undergo extensive differentiation and proliferation. Vascular endothelial growth factor (VEGF) and soluble factors, such as enzymes, cytokines, and growth factors, contribute to tissue modulation via angiogenesis, cell differentiation, and collagen thickening, resulting in skin rejuvenation and regeneration [3,6].

The addition of platelet-rich plasma (PRP) to fat grafts has been shown to increase graft survival. Upon activation, platelets release important growth factors. This improves fat graft vascularization and prevents adipocyte apoptosis and cutaneous trophicity in grafted areas [7]. Recent studies have shown that the addition of carbon dioxide (CO_2_) fractional laser to scar treatment improves efficacy [8].

Plastic surgery is an ever-evolving field; techniques that can be used to improve the results of fat grafting are constantly emerging. Despite their widespread use, there are no protocols demonstrating the efficacy of lipoconcentrate and nanofat grafting in combination with PRP, microneedling, and CO_2_ fractional laser in the treatment of facial scars and restoration of the skin’s natural condition. Thus, the purpose of this study was to evaluate the efficacy of this combination in achieving the desired esthetic and cosmetic results via skin regeneration and rejuvenation in the hope of developing a protocol in the future that may benefit patients with disfiguring and resistant scars after further trials.

## Case presentation

**Figure 1:**
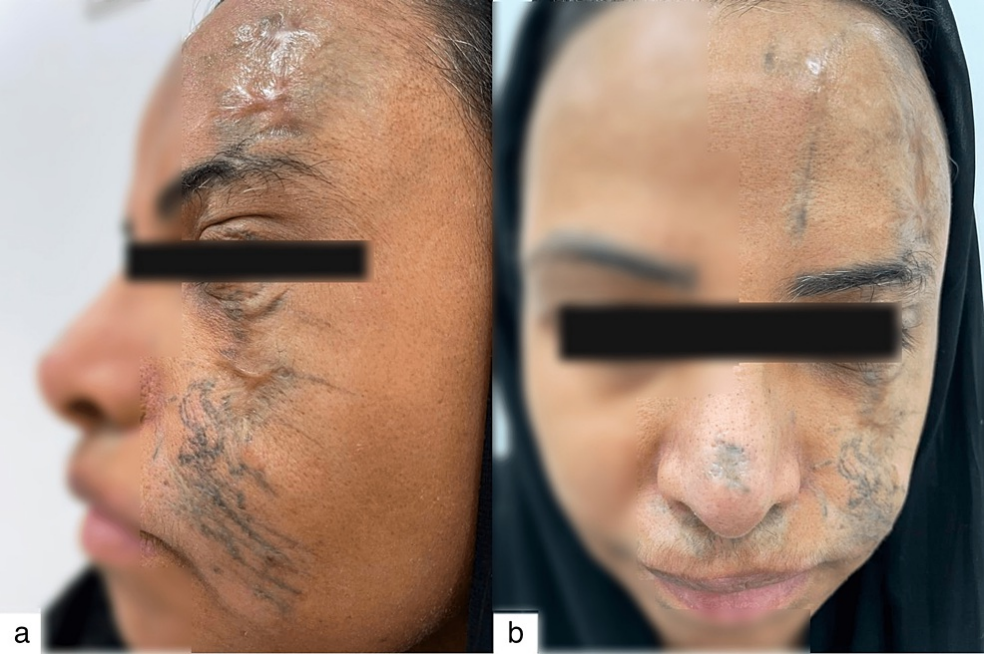
(a, b) A photograph taken prior to any intervention reveals asphalt particles embedded within the patient’s disfiguring scars as a result of a car accident. Additionally, there are depressed areas on the left cheek and forehead that contribute to the asymmetry.

A 53-year-old woman in good health was the subject of this case. She presented to the private clinic at Dr. Soliman Fakeeh Hospital in Jeddah, Saudi Arabia, complaining of traumatic facial injuries caused by a car accident that had occurred many years ago, burying asphalt particles in her facial scars and causing bluish discoloration of her skin. Among the numerous deep scars on the left side of the face was an atrophying scar on her left forehead extending up to her left eyebrow. In addition, she had numerous deep scars on her upper and lower eyelids, cheek, and nose, which caused fibrosis, contractures, and asymmetry (Figure 1).

This case report describes a patient who sustained facial trauma and was treated and monitored for three months. The patient was thoroughly evaluated, and we ensured that at least six months had passed for scar healing and that there were no active or immature scars, as scars tend to undergo an inflammatory remodeling phase. A consensus was reached to undergo surgery under general anesthesia for nanofat grafting using autologous lipoconcentrate. This was followed by three sessions of PRP and microneedling and then a session of CO_2_ laser fractional therapy. The patient presented with multiple facial complaints and sought both cosmetic and functional solutions. This evaluation is based on the plastic surgeon’s clinical assessment, image documentation, and patient satisfaction.

All procedures reported in this case report conformed to the ethical standards of the institution and the Declaration of Helsinki (as revised in 2013). The patient’s written informed consent was obtained.

Preparation and procedure technique

Fat Harvesting

Fat harvesting was the first step. In our case the abdomen was chosen as the donor site, but as described by Li et al. there is no difference in the quality of fat harvested from different areas [9]. Therefore, the choice of donor site can be made based on patient and surgeon preference.

A small vertical stab incision was made in the umbilicus. A tumescent solution was mixed from 1000 mL of normal saline containing 2 mL of 1% lidocaine and 0.1 mL of epinephrine in a 1:1000 ratio. Using a cannula attached to a 10-cc syringe, the tumescent solution was equally injected into both sides of the abdomen. The tumescent solution was allowed to rest for 10-15 minutes after injection to achieve optimal results before fat harvesting.

Microfat harvesting was conducted using a 2-mm St’rim multi-port microcannula (Wells Johnson company, Tuscan, AZ, USA) and 60-cc BD syringes (Becton, Dickenson and Company, Franklin Lakes, NJ, USA). Between 40 and 60 mL per side were aspirated manually to maintain adequate negative pressure. To minimize potential harm to the harvested fat cells, force-assisted liposuction was avoided. The aspirated fat was then transferred to syringes measuring 10 cc and 5 cc.

Fat Processing

Fat processing is a technique for purifying fat cells from blood and tumescent fluid. The harvested lipoaspirates were centrifuged at 1784 centrifugal force unit (g) for 3 minutes. This resulted in the clear separation of the fat, blood, and tumescent fluid. The lipoaspirates were then set aside in a syringe holder, and the supernatant (oil) and infranatant (blood) were discarded, leaving the middle layer containing the purified lipoaspirates.

**Figure 2:**
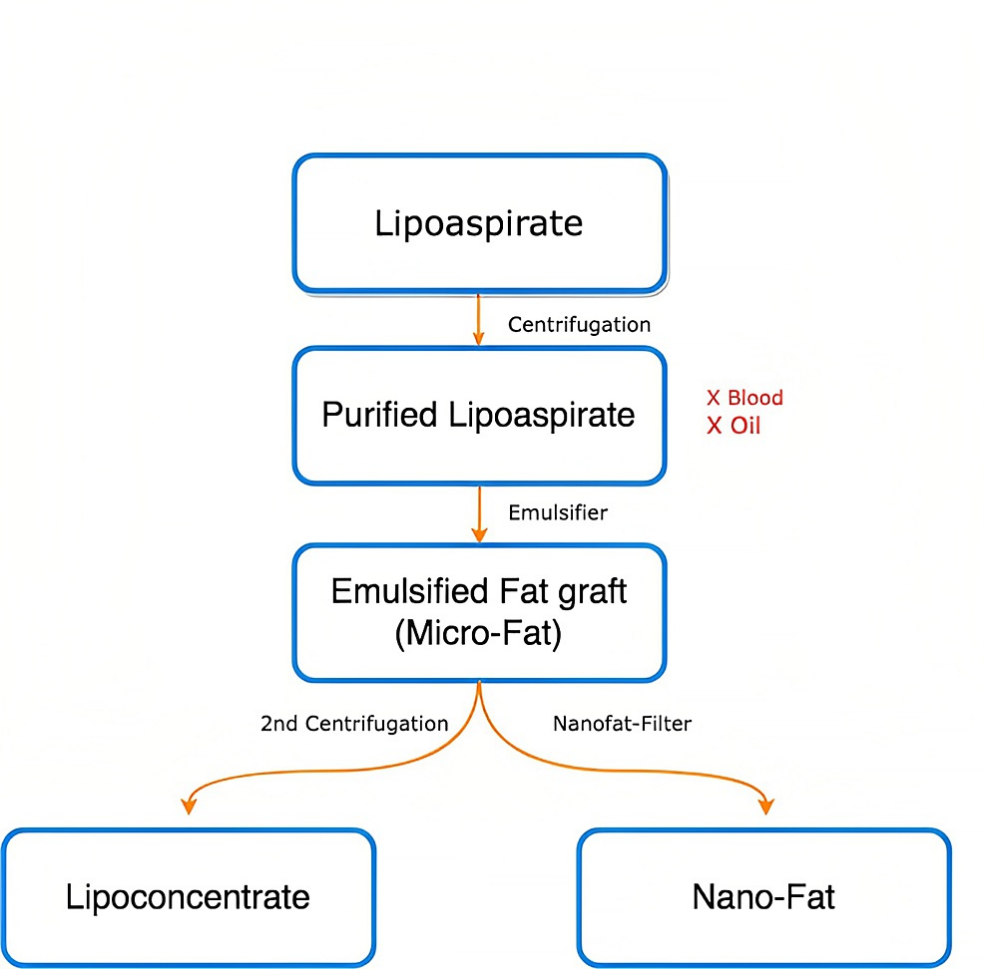
This diagram simplifies the different processing methods for harvested fat used in our article. The earliest products of harvested fats are lipoaspirates. After first centrifugation, they separate into three layers containing an oily layer, purified lipoaspirates, and blood with fluids. After removal of the blood and oily layer, emulsification of the purified lipoaspirates into microfat is achieved by passing them between two 10cc syringes linked with a Luer-Lok connector. From here this emulsion can be further processed into either nanofat by using a nanotransfer set or centrifuged for the second time creating lipoconcentrate.

These retained purified lipoaspirates contained microfat with the presence of viable adipocytes. To remove these viable adipocytes, the purified lipoaspirates underwent emulsification by passing them between two 10-cc syringes linked with a Luer-Lok (Xelpov Surgical, Houston, TX, USA) a total of 30 times. The microfat was then processed into nanofat and lipoconcentrate as simplified in Figure 2.

Lipoconcentrate Processing

After the emulsification of purified lipoaspirates into microfat, a second round of centrifugation at 1784 centrifugal force unit (g) for 3 minutes occurred. The second centrifugation again resulted in three layers, with the lipoconcentrate being the middle layer. Next, peanut sponges and gentle pressure on the syringe plunger were utilized to remove the top oily layer and bottom aqueous layer, respectively. The remaining lipoconcentrate consisted of a low-volume cell suspension and a high number of stromal vascular fraction (SVF) cells, including progenitor cells, which were then ready to be injected into the assigned areas.

Nanofat Processing

For nanofat processing, after emulsification of the purified lipoaspirates to microfat, the nanofat filtering system was used to isolate the ADCs. A set of Luer-Lok connectors was used for further emulsification of the microfat. First, a 2.4-mm Luer-Lok, then a 1.4-mm Luer-Lok, and a 1.2-mm Luer-Lok were used to shift the microfat between two 3-cc syringes for 30 passes. This turned the microfat into a liquid with a fine texture. The final step involved a single pass through the nano-transfer filter to ensure the isolation of ADCs without adipocyte survival. Because the nanofat transfer filter contains a single cartridge with a double filter, which only allows the passage of the smallest purified cell, the container can be clogged by tissues during the process. To prevent this, we changed the filter when resistance was felt to ensure the purity of the filtered fat cells.

Injection Techniques

The patient was informed of possible minor and major complications that could occur, including ecchymosis, pain, swelling, and infections, as well as major facial complications, usually vascular, that can lead to skin necrosis or blindness. To prevent devastating complications such as cerebral or ocular artery thrombosis, we ensured that there was no blood reflux into the syringe before injecting the fat graft into the face [10,11].

Lipoconcentrate injection technique: A 26-gauge needle was first used to create entry points for the 0.8-mm needle. The lipoconcentrate was loaded into 3-cc syringes for injection. A rigotomy was performed at the areas of injection to release fibrosis, reduce tension, and decrease scar contraction. A 0.8-mm cannula was then carefully advanced through the subcutaneous tissue until it reached the distal end of the area to be grafted. Prior to injecting the fat graft into the face, we ensured that no blood refluxed into the syringe. Using a fanning technique, the lipoconcentrate was subcutaneously injected in small packets of 0.5 cc along the scar, until the appropriate volume was reached, and the target areas were filled. The left eyelid, forehead, and cheek were treated with lipoconcentrate to provide a controlled amount of volume in depressed areas on the left side of the face. A small amount was also applied laterally to the right eye.

Nanofat rejuvenative injection technique: Before nanofat injection, rigotomy was performed to improve scar pliability and make them less evident. The nanofat was transferred to 3-cc syringes and then injected through a 30-gauge needle for superficial intradermal injections. In our case, the intradermally injected nanofat was 1-2-cc per scar area all over the face, focusing primarily on the areas of discoloration and fibrosis. This was done to promote skin regeneration, rejuvenation, and softening. The same fanning and retrograde injection technique was used, and a yellowish discoloration of the skin was visible upon injection. The Hydra 20 microneedle titanium applicator (FACE Med store, New York, NY, USA) bottle was then used to inject nanofat superficially into multiple areas to intensify the regenerative results.

In most cases, injections are made subcutaneously just below the scar tissue, and the injected fat is usually partially resorbed over time. Therefore, all injection areas intended for correction were overfilled. Clinical evaluation of the injected areas was performed by assessing the overlying skin tension and whitening.

PRP and Microneedling

First, 10-20 mL of blood was drawn from the median cubital vein. The tubes were rotated in a centrifuge machine at 1784 centrifugal force unit (g) for 5 minutes. This separated the blood into three layers: the red blood cells (RBC) layer (bottom), the buffy coat (middle), and the PRP (top). The PRP was collected in a new tube and then loaded into 1-cc syringes. This process was done under aseptic conditions.

A disposable Hydra 20 microneedle titanium applicator with an 8-mL vial was used to pump microdroplets of PRP after each tapping motion. The device’s lid contained twenty 1.5-mm needles to achieve deep penetration of the skin, allowing for better absorption. Hydra-microneedling was done while the patient was under general anesthesia in the operating room after fat grafting and lipofilling were completed. The patient’s skin was first cleaned with ethyl alcohol to remove all oils on the skin surface, and then PRP was injected through the Hydra microneedle using gentle manual pressure and release. PRP microneedling was then repeated post-operatively in the outpatient clinic using a Dermapen device (Dr. Pen, Perth, Australia) consisting of a spring-loaded needle tip containing 11 ultra-fine sterile microneedles, which were injected into the skin to create tiny punctures intradermally. The PRP collected using the same technique mentioned above was then applied on top of the punctured areas to allow deeper penetration and absorption of the PRP. This was repeated twice post-operatively at four-week intervals.

CO_2_ Fractional Laser

One month after completing the final PRP and microneedling session and four months after her initial fat grafting operation, she underwent a single session of ablative fractional CO_2_ laser therapy (wavelength of 10.6 μm) at a specialized laser center. The parameters used in static mode were a density of 50 spots/cm^2^, a power of 30 watts, and a beam tip size of 120 μm. The ablative fractional CO_2_ laser includes the Controlled Chaos Technology feature, which places maximum distance between successive beams. The use of this technology increases safety, reduces postoperative discomfort, and results in a faster recovery period.

Post-operative Care and Follow-up Assessment

The patient was instructed to apply a fusidic acid ointment topically every day for three days after surgery and following each PRP microneedling session. In order to maximize the potency and efficacy of the grafted fat and injected PRP, she was also instructed to avoid compression and cooling of the area. Massaging of the scar was to begin approximately six weeks after surgery. She was evaluated every four weeks for four months following treatment.

Results

We used a combined approach for scar management and skin enhancement involving nanofat and lipoconcentrate grafting, PRP, microneedling, and CO_2_ fractional laser. Our preoperative assessment of the scars ensured that over six months had passed since they had developed and that no active or immature scars were present.

**Figure 3:**
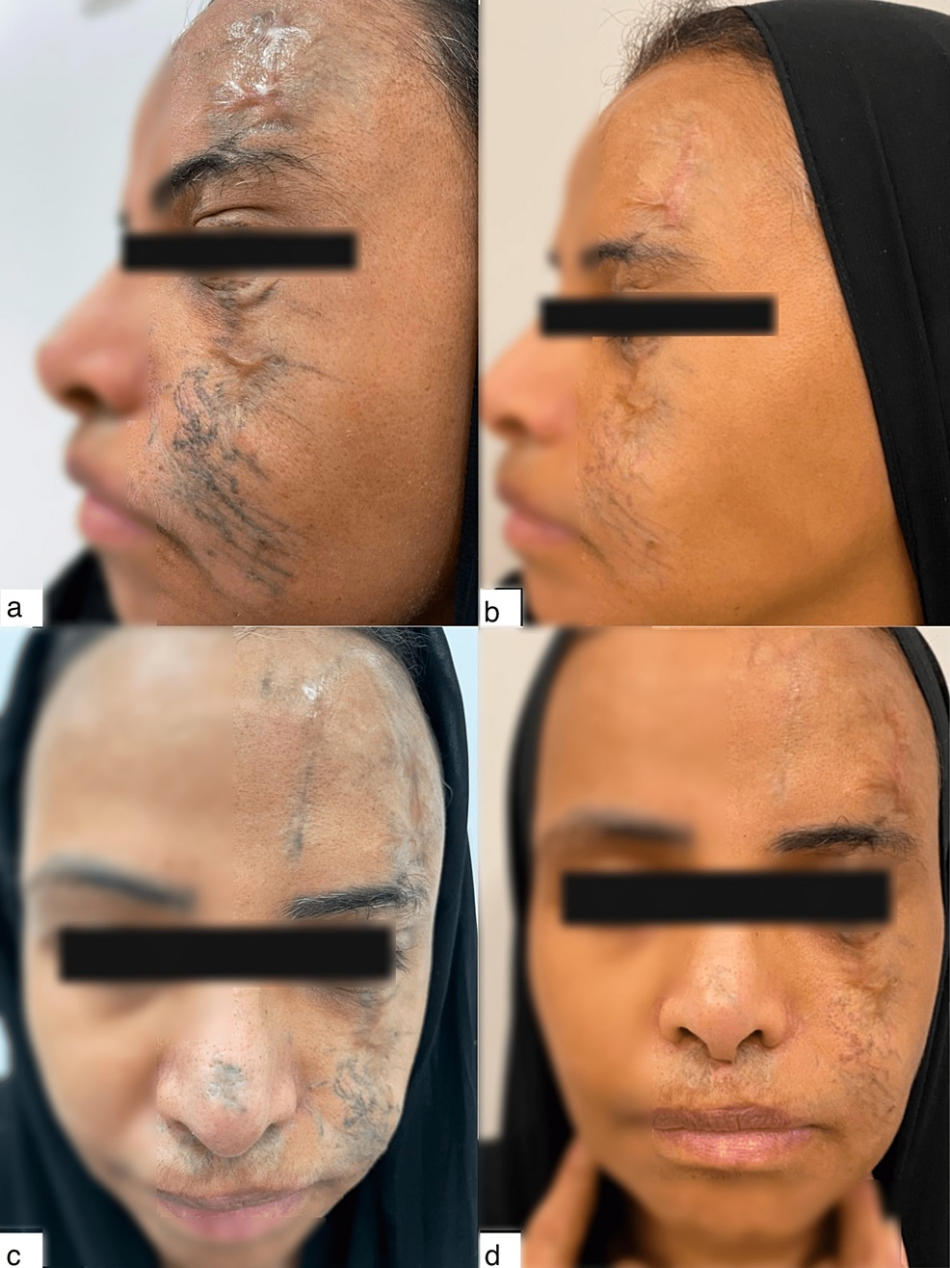
(a, c) Photos taken before the start of treatment. (b, d) Photos taken after nanofat and lipoconcentrate fat grafting, three PRP microneedling sessions, and a session of CO2 fractional laser showing tremendous results mainly in terms of skin color and scar size and texture. Volume enhancement and correction of facial asymmetry can also be observed.

At the end of the four-month treatment plan, including the fat grafting procedure, three PRP and microneedling sessions (one month between each session), and CO_2_ fractional laser therapy, the patient’s skin appearance, color, and texture had improved significantly. Volume enhancement had corrected the depressions and asymmetry she had previously complained of. She was very satisfied with the results (Figure 3).

## Discussion

Psychological and psychosocial effects of facial complaints contribute to the growing popularity and significance of esthetic surgery. This is especially true for scars resulting from accidents or burns. Scars are associated with decreased self-esteem, stigmatization, disruption of daily activities, anxiety, and depression. Therefore, the demand for surgical correction has increased [12,13].

Historically, facial esthetic treatment was restricted to non-operative versus surgical approaches. Non-surgical treatments are transient and must be repeated to maintain results. For difficult hypertrophic scars, these include topical treatments, lasers, fillers, chemical peels, and steroid injections. Surgical management, in contrast, is permanent but invasive and expensive. These options were historically restricted to scar revision and potential facelift procedures [14].

The development of regenerative therapies has revolutionized esthetic facial surgery. The constant development of technology and the publication of scientific findings have made autologous fat grafting feasible [15-22]. This has led to significant improvements in skin regeneration, scar healing, and facial cosmetic surgery outcomes.

Fat grafts can address various facial concerns. The type of fat graft utilized depends on the ailment being treated [3,5]. Multiple studies have demonstrated that nanofat grafts are the richest source of ASCs, which promote angiogenesis, modulate collagen deposition, and prevent fibrosis. In addition, ASCs produce growth factors and cytokines that reduce inflammation and increase the turnover of the extracellular matrix. Consequently, they enhance wound healing, skin texture, and complexion without adding volume [23-28]. Lipoconcentrates contain a small quantity of adipocytes in addition to ASCs, making them ideal for skin rejuvenation with controlled volume enhancement [3].

Nanofat grafting and lipoconcentrates have been shown to improve scar regeneration and skin rejuvenation, among other facial issues [3,29,30]. Zheng et al. [31] concluded that co-transplantation of nanofat with macrofat grafts improved fat graft survival via proangiogenic, antiapoptotic, and pro-proliferative effects on ADCs. Similar outcomes were observed in previous studies involving the combination of nanofat grafts with dermal scaffolds, highlighting the role of nanofat-rich ASCs in enhancing regenerative outcomes [15].

PRP is a natural reservoir for many growth factors, including platelet-derived growth factor and VEGF. In addition to increasing cell proliferation and differentiation, it also stimulates angiogenesis and collagen production, both of which trigger the natural process of skin regeneration, healing, and repair as well as fat graft survival [32-35]. It is safe due to its autologous nature as well as time and cost-effective due to its ease of extraction and preparation. Lei et al. [36] tested four combinations of PRP with fat/nanofat and normal saline with fat/nanofat and injected them into mice. Twenty male nude mice were divided into four groups: PRP/fat, PRP/nanofat, normal saline (NS/fat), and NS/nanofat. After one and three months, the grafts were extracted and examined. Higher graft weights were found in the PRP/fat group, while a higher degree of neovascularization and the expression of VEGF was found in the PRP/nanofat group, demonstrating the effect of PRP and nanofat on wound healing and rejuvenation. Cervelli et al. [34] reported excellent esthetic results and patient satisfaction when combining PRP with a purified fat graft to correct facial aging by correcting volume loss, wrinkles, and increasing elasticity. As a solution for severe acne scars, Pons et al. [35] used a mixture of nanofat and PRP, showing improved skin elasticity and scar reduction.

Microneedling has gained popularity as a minimally invasive esthetic technique to treat skin aging, scarring, striae, and hair loss, among other indications [37-39]. Microneedling can deliver PRP intraepidermally or intradermally [40], increasing PRP absorption, which has shown better clinical results post-scarring [37]. No studies in the literature have described the optimal number of microneedling and PRP sessions. From our clinical experience, the number of sessions depends on the patient’s scars and their body’s response to treatment. Sessions should be performed monthly with a minimum of three sessions, and an additional session can be scheduled for maintenance therapy six weeks after the last session.

A further method of enhancing scar treatment and skin rejuvenation was the addition of a single session of CO_2_ fractional laser, which has been used as a monotherapy for scar treatment for decades [41]. Compared to CO_2_ fractional laser monotherapy, Huang et al. [26] found that the combination of CO_2_ fractional laser and autologous fat for the treatment of hypertrophic scars had a greater synergistic effect that improved symptomatic and healing outcomes.

This case study demonstrates the synergistic effects of lipoconcentrate and nanofat grafting, PRP microneedling, and CO_2_ fractional laser in facial esthetic surgery. Plastic surgeons at Dr. Soliman Fakeeh Hospital determined the outcomes based on patient satisfaction, image documentation, patient evaluation, and follow-up. This study illustrates the diversity of fat graft processing and injection techniques, highlighting their adaptability. In addition, we discovered that the combination of the aforementioned techniques produced superior results to monotherapy in the treatment of severely disfigured scars.

Despite reports that lipoconcentrate and nanofat grafting, PRP, microneedling, and CO_2_ fractional laser can individually treat a variety of facial esthetic problems, there is little evidence to suggest that these techniques should be combined to treat scars, asymmetry, and skin rejuvenation. This case report was motivated by the paucity of research regarding the use and benefits of this combined therapy, particularly in difficult-to-treat and disfiguring scars. This report demonstrates basic clinical methods and outcomes for future research purposes.

Our objective was to apply these techniques to our patient, observe their synergistic effect on a variety of facial cosmetic complaints, and demonstrate their efficacy. This combined therapy was observed to improve normal skin tissue as well as scar and fibrotic tissue, resulting in an improvement in the appearance of volume loss, facial depression, and asymmetry. However, precise quantitative data and criteria for evaluating patient improvement throughout treatment are limited. In addition, further research is required to develop a protocol that achieves optimal results, including the optimal number of PRP, microneedling, and laser sessions; the duration between sessions; and the need for maintenance therapy after fat grafting. A case series could also be conducted in the future to evaluate the synergistic effects on various patients and affirm its efficacy.

## Conclusions

This study demonstrates the efficacy and safety of combination therapy of nanofat and lipoconcentrate fat grafts, along with three PRP, microneedling sessions, and a session of CO_2_ fractional laser as an approach to treating a variety of facial complaints, including volume loss, scars, contractures, and skin rejuvenation. Therefore, we highly recommend the techniques presented in our study for cosmetic, aesthetic, and regenerative facial skin care. To confirm these results and create a standardized protocol that could be used to treat patients, further research and treatments of different patients must be carried out.
